# Observations on Sun-Stroke, in a Letter to the Editor

**Published:** 1855-12

**Authors:** B. M. Wible

**Affiliations:** Louisville, Ky.; Jefferson St.


### MEDICINE AND SURGERY.

DECEMBER, 1855. NEW SERIES. VOL. IV.—NO. 6.

### ART. I.—OBSERVATIONS ON SUN-STROKE, IN A LETTER TO THE EDITOR.

BY B. M. WIBLE, M. D., OF LOUISVILLE, KY.

THE following observations and reflections upon *sun-stroke*, I had intended to present to the Medical Club, but as I was absent from the city at the time when that subject was discussed before it, I will now ask for them a place, Mr. Editor, in the columns of your Journal:

The majority of the cases of sun-stroke which have come under my observation, occurred during the exceedingly warm weather of the summer of 1855. The following are the phenomena which were particularly observed: 1. Great heat of the surface persisting in fatal cases for some time after death. In one to which I was called, and where I found the patient dead, the skin seemed to be as warm as we find it in the hot stage of an intermittent. 2. Loss of muscular power—the patient sliding down in bed, and the arms and legs lying powerless and flabby. 3. After the muscular power becomes somewhat restored, in some cases, a kind of spasm or convulsion came on, attended with a sort of phrenzy.

These are the prominent features of the disease which arrested my attention. I have no doubt that where careful observation will have been made, that in all cases where the muscular power has been suddenly overcome, the pupils will be found dilated; in the convulsive stage, alternately dilated and contracted; and in the comatose stage following the convulsions, rigidly contracted.

The cause of the disease is, I think, *over-stimulation;* for I have known it to occur in those only who have been exposed to a high atmospheric temperature, after the free use of stimulating food and drinks. These conjoined stimulants exhaust the nervous excitability; the medulla oblongata, losing its excitability, no longer responds to the excitor impressions of respiration—the breathing of course ceases, and the patient dies. If death occurred as the direct effect of nervous exhaustion, I should not expect to find any post-mortem appearances indicative of the disease; but if it occurred after the convulsions and coma, I should expect cerebral congestion as the *effect* of the convulsions.

The treatment of the first stage of the disease, or that of nervous exhaustion, ought to be conducted as follows: The patient should be kept in a state of absolute repose, and the temperature of the body gradually reduced by a cool and airy room, and the gentle application of cold water to the surface. From theoretical, and from what actual observation has taught me, I would advise the administration of a tea spoonful of the tincture of opium as soon as it could be given. Absolute freedom from all disquieting agents is of the utmost importance, because every muscular effort, every excitation of the system, the action of every arterial stimulant, further exhausts the diminished nerve force. At any time that the breathing should cease, I would advise a resort to artificial respiration, and to make this effectual, it might be necessary to inflate the lungs through an artificial opening made into the trachea. The convulsive or comatose stage would be best treated by an emetic of ipecacuanha, and by cold applications to the head. Blood-letting in some cases might be necessary.

It is difficult to explain why exhaustion of the vis nervosæ should lead to convulsions, but the fact is well known. The nervous exhaustion which characterizes sun-stroke is like that produced by chloroform, and the restoration from it, when produced by either cause, is sometimes attended by convulsive phenomena.

*Jefferson St., Dec.,* 1855.

